# Help-seeking intentions for early signs of mental illness and their associated factors: comparison across four kinds of health problems

**DOI:** 10.1186/s12889-016-2998-9

**Published:** 2016-04-07

**Authors:** Machi Suka, Takashi Yamauchi, Hiroki Sugimori

**Affiliations:** Department of Public Health and Environmental Medicine, The Jikei University School of Medicine, 3-25-8 Nishi-Shimbashi, Minato-ku, Tokyo, 105-8461 Japan; Center for Suicide Prevention, National Institute of Mental Health, National Center of Neurology and Psychiatry, Tokyo, Japan; Department of Preventive Medicine, Graduate School of Sports and Health Sciences, Daito Bunka University, Saitama, Japan

**Keywords:** Help-seeking, Mental health, Questionnaire survey

## Abstract

**Background:**

Failure and delay in initial treatment contact for mental disorders has been recognized as an important public health problem. According to the concept of mental health literacy, recognition of symptoms is crucial to making decisions to seek or not seek professional help. The aims of this study were to investigate the types of health problems for which Japanese adults intend to seek help, their preferred sources of help, and the factors associated with help-seeking intentions.

**Methods:**

A cross-sectional web-based survey was conducted in June 2014 among Japanese adults aged 20–59 years. A total of 3308 eligible respondents were included in this study. Help-seeking intentions were measured by listing potential sources of help (including ‘would not receive help’) and asking which ones would be chosen in four health conditions indicated by irritability, dizziness, insomnia, and depressed mood, respectively.

**Results:**

In the case of dizziness, 85.9 % of the participants reported a positive help-seeking intention and 42.7 % gave first priority to seeking help from formal sources. These percentages were smaller in the cases of insomnia (75.4 and 25.0 %), depressed mood (74.9 and 18.7 %), and irritability (72.9 and 0.9 %). Multiple logistic regression analysis revealed that the factors significantly associated with help-seeking intentions were almost identical across the four health problems. In particular, perception of family and friends regarding help-seeking, psychiatric history, contact with people with mental illness, better health literacy, and neighborhood communicativeness were significantly associated with the overall help-seeking intention and also the help-seeking intention from formal sources for all the problems of dizziness, insomnia, and depressed mood.

**Conclusions:**

The majority of participants indicated their intentions to seek help, but psychological problems (insomnia and depressed mood) were less likely to induce help-seeking intentions than a physical problem (dizziness). Besides developing health literacy skills, community-based interventions for creating a friendly approachable atmosphere and facilitating daily interactions with family, friends, and neighbors may be worth considering as a possible public health strategy for encouraging help-seeking whether for psychological or physical problems.

## Background

The Global Burden of Disease Study 2013 [1] revealed that mental and substance abuse disorders accounted for 21.2 % of years lived with disability (YLDs). Major depressive disorder was a crucial contributor in both developed and developing countries: it is the leading cause of YLDs in 56 countries, the second leading cause in 56 countries, and the third leading cause in 34 countries. There is now a variety of effective treatments available for mental disorders. To prevent negative sequelae of mental disorders, prompt initial contact with healthcare providers is needed after first onset of symptoms. However, many affected individuals in both developed and developing countries delay in seeking professional help and fail to receive effective treatment [2, 3].

Jorm et al. have coined the term “mental health literacy” with the definition “knowledge and beliefs about mental disorders which aid their recognition, management or prevention” [4]. According to the concept of mental health literacy, recognition of symptoms is crucial to making decisions to seek or not seek professional help [5]. Signs and symptoms of mental illness include a variety of psychological as well as somatic symptoms. As for patients with depression in primary care, approximately two thirds of the patients present with somatic symptoms [6]. People who begin to exhibit a symptom of mental illness should recognize the early signs and take prompt action to recover their own health. Unfortunately, a number of population surveys have revealed that many people cannot correctly recognize symptoms depicted in a vignette as a mental disorder [5]. If people do not recognize the signs and symptoms of mental illness, they will not seek help, and thus healthcare services will be ineffective regardless of their availability.

People who should receive professional help are not limited to those who meet the diagnostic criteria from the Diagnostic and Statistical Manual of Mental Disorders (DSM). In order to encourage early help-seeking for mental illness, policymakers need to understand what kinds of early signs of mental illness are less likely to induce help-seeking behavior. Previous studies have revealed that the majority of patients who were diagnosed with major depressive disorder had reported only somatic symptoms as the reason for visiting the physician [6]. This indicates that patients with depression in primary care tend to report somatic symptoms more readily than they report psychological symptoms. Unfortunately, most of the previous studies have been conducted among clinical populations, i.e. patients who visited their physicians to seek help. It has been reported that the likelihood of seeking help varied depending on the type of disorder [7, 8]. However, to our knowledge, there have been no attempts to identify differences in seeking help for different kinds of symptoms of mental illness. It remains unknown how much more likely people are to seek help for somatic symptoms as compare to psychological symptoms. Such symptom-based approaches may provide useful information for dealing with the problem of failure and delay in initial treatment contact after first onset of symptoms.

Japan has achieved universal health coverage, which provides relative equality of access to healthcare services [9]. Despite having the universal healthcare system, failure and delay in initial treatment contact for mental disorders has been recognized as an important public health problem in Japan [2, 3]. A comparative survey between Japan and the United States [10] indicated that Japanese people exhibited greater reluctance to seek professional help. A comparative survey between Japan and Australia [11] indicated that Japanese people were more reluctant to use psychiatric labels, more reluctant to discuss mental illness with others outside the family, and less positive about the benefits of seeking professional help. An international study as well as other cross-cultural comparative studies suggested that the experience of symptoms varies little across countries and cultures, whereas the reporting of symptoms can be influenced by sociocultural factors, particularly stigma surrounding mental illness [12–14] These findings suggest that the public beliefs about mental illness and its treatment may contribute to failure and delay in initial treatment contact. Unfortunately, only a few studies have addressed help-seeking behavior of Japanese people, [15–17] and very little is known about the barriers and facilitators of seeking professional help in Japan. Previous studies in different countries have proposed a variety of factors that may influence help-seeking behavior for mental illness, including physical dysfunction, [18] psychological distress, [18] exposure to mental illness, [19–21] knowledge about mental illness, [22] stigmatizing attitudes to mental illness, [20, 21] perceived effectiveness of professional help, [23] social network, [24] and neighborhood context [25] In order to develop public health strategies for encouraging help-seeking for mental illness, policymakers need to have a clear understanding of help-seeking behavior of their target population groups and to identify the factors that will have a significant impact on their help-seeking behavior.

The aims of this study were to investigate the types of health problems for which Japanese adults intend to seek help, their preferred sources of help, and the factors associated with help-seeking intentions. The study participants were asked their intentions to seek help in four health conditions indicated by irritability, dizziness, insomnia, and depressive mood, respectively. A variety of factors mentioned above were examined for their associations with help-seeking intentions. In contrast to previous studies that examined help-seeking intentions for specific mental disorders, [7, 8] this study focused on early signs that could appear before it is officially diagnosed as major depressive disorder [6, 26]. Depressed mood and insomnia are included in the DSM-5 diagnostic criteria for major depressive disorder [26]. Dizziness and irritability are not part of the diagnostic criteria but are most commonly reported symptoms in patients with depression in primary care [6]. A follow-up study of Japanese workers suggested that dizziness, as well as loss of interest, may be a significant predictor of depression [27]. Recent studies on the significance of irritability in major depressive disorder in adults suggested that irritability may serve as a severity marker, [28, 29] or a subtyping distinction [29, 30]. We believe the findings of this study will provide a new direction for public health strategies for encouraging help-seeking for mental illness as well as contribute to a better understanding of help-seeking behavior of Japanese people.

## Methods

### Participants

A cross-sectional web-based survey was conducted in June 2014 among Japanese adults aged 20–59 years. Details of the survey have been presented elsewhere [31]. Briefly, participants in the survey were recruited from an online research panel of a a leading research company in Japan (INTAGE, INC., Tokyo, Japan). Students and medical professionals were excluded from recruitment, because their attitudes to help-seeking seem different in kind from that of lay people [32, 33]. Recruitment e-mails were sent to 8721 randomly selected eligible registrants, and applicants for participation in the survey were accepted in the order of receipt until the number of participants reached the quotas (100 people for each age/gender/prefecture stratum). All participants voluntarily agreed to participate in the survey after reading a description of the purpose and procedure of the survey. Consent to participate was implied by the completion and submission of the survey.

A total of 3365 responses were obtained over 8 days of recruitment. Of these, 51 people provided incomplete or inconsistent answers to questions. Six people reported having the following diseases that affected their activities of daily living at the time of the survey: stroke, epilepsy, syringomyelia, multiple sclerosis, nerve palsy, and chronic thyroiditis. People with such diseases were excluded because of how these diseases can impair help-seeking decision making by presenting practical barriers to access and utilization of healthcare services. The remaining 3308 participants were finally included in this study. Table 1 shows the characteristics of the study participants. According to the 2010 national census, [34] the percentage of the Japanese population aged 20–59 years with university degrees was 21.9 %, considerably lower than that of this study (40.4 %), whereas the percentages of married and employed population were 58.2 and 72.7 %, respectively, almost equal to that of this study (59.3 and 76.7 %, respectively).

**Table 1:** Characteristics of the study participants

		N	
Gender	Male	1621	49.0 %
	Female	1687	51.0 %
Age	20–29 years	797	24.1 %
	30–39	842	25.5 %
	40–49	837	25.3 %
	50–59	832	25.2 %
Education	High school	1066	32.2 %
	Junior college/vocational school	905	27.4 %
	University/graduate school	1337	40.4 %
Marital status	Married	1960	59.3 %
	Unmarried	1184	35.8 %
	Divorced/widowed	164	5.0 %
Occupation	No occupation	770	23.3 %
	Temporary or part-time job	560	16.9 %
	Full-time job	1978	59.8 %
Household income	<2.0 million yen ^a^	363	11.0 %
	2.0–3.9 million	777	23.5 %
	4.0–5.9 million	937	28.3 %
	6.0–7.9 million	618	18.7 %
	8.0–9.9 million	347	10.5 %
	10.0+ million	266	8.0 %
Medical condition	No disease	2449	73.6 %
	Any disease	879	26.4 %

^a^ One million yen was about 10,000 U.S. dollars at the time of the survey

The study protocol was approved by the ethics committee of the Jikei University School of Medicine and has been conducted in accordance with the Ethical Guidelines for Epidemiological Studies by the Japanese Government.

### Measures

The questionnaire asked about problem-specific help-seeking intentions, medical conditions, psychological well-being, exposure to mental illness, health literacy, stigmatizing attitudes to mental illness, perceived effectiveness of professional help, social network, and neighborhood context. The eight factors that may influence help-seeking behavior for mental illness were derived from the literature [18–25]. The components of the questionnaire relevant to this study are detailed below.

#### Problem-specific help-seeking intentions

A literature review [35] reported that help-seeking has been defined and measured inconsistently across studies in the mental health context. In the absence of consensus on definition and measurement of help-seeking, intentions to seek help for mental health problems are often measured by listing potential sources of help and asking which ones would be chosen in a specified health condition [35–38]. This commonly used methodology was adopted for this study.

Participants were asked what they would do if they were in four health conditions indicated by irritability, dizziness, insomnia, and depressed mood, respectively: every day for more than 2 weeks, 1) you were irritated by trouble in your neighborhood (irritability); 2) you had severe dizziness, often accompanied by nausea (dizziness); 3) you could not sleep well at night despite being tired (insomnia); and 4) you felt so depressed all day that it was difficult to function (depressed mood). According to the DSM-5 diagnostic criteria for major depressive disorder, [26] the time period of 2 weeks was applied to all the health problems. Table 2 shows an overview of the four health problems presented in the questionnaire. The four health problems were designed so as to have different features. Two psychological symptoms and two somatic symptoms were selected from among most commonly reported symptoms in patients with depression in primary care [6]. Compared to depressed mood and insomnia that are included in the DSM-5 diagnostic criteria for major depressive disorder, [26] dizziness is more often caused by physical problems [39]. The irritability problem of this study was supposed to be triggered by an obvious non-medical problem (i.e. neighborhood trouble), so that help from medical professionals was not always necessary for this health problem. Our recent study revealed that tendency to consult about everyday affairs is a key player in help-seeking decision-making for mental illness [17]. Participants who reported a positive help-seeking intention for the irritability problem could be considered as those having a tendency to consult about everyday affairs.

**Table 2:** Overview of the four health problems presented in the questionnaire

Health problem	Type of symptom	Most probable cause (or trigger)
Irritability	Psychological symptom	Non-medical problem
Dizziness	Somatic symptom	Physical problem
Insomnia	Somatic symptom	Mental problem
Depressed mood	Psychological symptom	Mental problem

All four health problems were presented to each participant one at a time in random order. Respondents chose all agreeable options for each health problem and also marked the option that they gave first priority. The options were 1) consult with my family or relative, 2) consult with my friend, 3) consult with my colleague, 4) see a health professional at my workplace, 5) see a medical professional at any hospital, 6) use a common consultation service provided by a public or private institution, 7) seek advice on the Internet, 8) seek advice from the other sources, 9) would not receive help from others because I felt it impossible to seek help for some reason, and 10) would not receive help from others because I feel no need for help. To prepare the potential sources of help options, we reviewed the literature relevant to this topic and selected appropriate ones in consideration of Japanese healthcare system [9]. We sought comments on the draft version from some lay people and then completed the final version.

In addition to their intentions to seek help, participants were asked to rate how likely their family and friends would think that they should 1) receive help from others and 2) see a medical professional, respectively for each health problem.

In reply to the question about their help-seeking intention, those who chose the options of 4)-5) were counted as having positive help-seeking intentions from formal sources, and those who chose the options of 1)-3) 6)-8) were counted as having positive help-seeking intentions from informal sources. Those who chose the options of 9)-10) were counted as having no help-seeking intention, and they were additionally asked the reason why they would not seek help from others for the health problem.

#### Medical condition

Participants were asked to report whether they had any chronic disease for which they were undergoing medical treatment. The list included hypertension, diabetes, dyslipidemia, stroke, heart trouble, renal failure, cancer, insomnia, depression, and others.

#### Psychological well-being

The World Health Organization Five Well-Being Index (WHO-5) [40, 41] was used to measure psychological well-being. Respondents choose one of 6 options (scored 0-5 points) in response to each of the 5 statements. The WHO-5 score is calculated by totaling the scores of the five responses, ranging between 0 and 25 points with high scores indicating better well-being.

#### Exposure to mental illness

Psychiatric history was assessed using the following question: have you ever seen a medical professional for your mental health problem? Those who gave an affirmative response were counted as having used mental health services.

The Reported and Intended Behaviour Scale (RIBS) [42] was used to determine the extent of contact with people with mental illness. The first subscale consists of four questions about living with, working with, living nearby, and having a close friendship with people with mental illness, either at present or in the past. Those who answered ‘yes’ to at least one question were counted as having had contact with people with mental illness.

#### Health literacy

Health literacy is originally defined as the cognitive and social skills which determine the motivation and ability of individuals to gain access to, understand, and use information in ways which promote and maintain good health [43]. On the basis of our previous findings that people with adequate health literacy tend to choose healthy behavior, [44] we hypothesized that people with adequate health literacy are more likely to seek help for any health problem (whether for physical or mental disorders). Suka et al. developed a generic health literacy measure for Japanese adults, named the 14-item Health Literacy Scale (HLS-14) [45]. Because of dealing with various types of health problems, we decided to use the generic health literacy measure rather than specific measures in this study.

The HLS-14 consists of 5 items for functional literacy, 5 items for communicative literacy, and 4 items for critical literacy. Respondents choose one of 5 options (scored 1–5 points) in response to each of the 14 statements. The HLS-14 score is calculated by totaling the scores of the 14 responses, ranging between 14 and 70 points, with high scores indicating better health literacy.

#### Stigmatizing attitudes to mental illness

Referring to the questionnaire for the Eurobarometer 64.4 survey, [46] four stigmatizing attitudes to mental illness were assessed using the following statements, respectively on a 4-point scale: people with mental illness 1) constitute a danger to others (dangerous); 2) are unpredictable; 3) have themselves to blame (blameworthy); and 4) never recover. For reference, the Cronbach alpha of this scale was 0.68 in this study, which reached the minimally acceptable level of internal consistency (≥0.65) [47]. For analysis, the responses were dichotomized into positive (strongly agree/agree) and negative (disagree/strongly disagree).

#### Perceived effectiveness of professional help

Referring to the questionnaires for the World Mental Health Survey (WMH) Initiative Version of the World Health Organization (WHO) Composite International Diagnostic Interview (CIDI) Part II, [48] perceived effectiveness of professional help was measured using the following two questions: 1) of the people who see a professional for serious mental illness, what percent do you think are helped? (range 0–100 %); 2) of those with serious mental illness who do not get professional help, what percent do you think get better even without it? (range 0–100 %). The responses to the first question showed a normal distribution with a mean of 48.2 (SD 23.6) and a median of 50. The responses to the second question showed a right-skewed distribution with a mean of 27.0 (SD 21.0) and a median of 20. To obtain a single index value for perceived effectiveness of professional help, the percentages on the two questions were subtracted (the first question minus the second question), with high values indicating more positive perception of the effectiveness of professional help [17, 23, 31]. The calculated values, showing a left-skewed distribution with a mean of 21.2 (SD 29.7) and a median of 20, were trichotomized into positive (1 < %, better than no help), neutral (0 %, equal to no help), and negative (<-1 %, worse than no help). Participants were also asked whether they would be embarrassed if their friends knew they were getting professional help for mental illness.

#### Social network

The abbreviated Lubben Social Network Scale (LSNS-6) [49, 50] was used to measure social network. This scale consists of 3 questions for family ties and 3 questions for friendship ties. Respondents choose one of 6 options (scored 1–6 points) in response to each of the 6 questions. The LSNS-6 score is calculated by totaling the scores of the 6 responses, ranging between 6 and 36 points, with high scores indicating greater ties to family and friends.

#### Neighborhood context

Neighborhood is characterized as a geographically localized community often with face-to-face interactions among members. Referring to the questionnaire for the Health Survey of People Affected by the Great East Japan Earthquake, [51] four specific features of neighborhood context relevant to neighborhood social capital were assessed using the following statements, respectively on a 5-point scale: people in your neighborhood 1) say hello whenever they pass each other (communicative); 2) trust in each other (trustful); 3) help each other (helpful); and 4) work together to solve neighborhood problems (cooperative). For reference, the Cronbach alpha of this scale was 0.87 in this study, which reached the minimally acceptable level of internal consistency (≥0.65) [47]. For analysis, the responses were dichotomized into positive (strongly agree/agree) and negative (not sure/disagree/strongly disagree).

### Statistical Analysis

The percentages of participants reporting a positive help-seeking intention for each health problem were compared using Chi-square test. McNemar test was used to assess the significance of the difference between the two correlated proportions. Multiple logistic regression was used to identify factors independently associated with the help-seeking intention for each health problem. Adjusted odds ratios (ORs) and 95 % confidence intervals (CIs) were calculated for the overall help-seeking intention (whether from formal or informal sources) and that limited to formal sources, respectively. The analysis of help-seeking intention from formal sources was not performed for irritability, because help from formal sources seems not always necessary for this health problem. All statistical analyses were performed using SAS ver.9.4 software (SAS Institute, Cary, NC). Significant levels were set at *p* < 0.05.

## Results

Table 3 shows the percentages of participants reporting a positive help-seeking intention for the four health problems. In the case of dizziness, 85.9 % of the participants were recognized as having a positive help-seeking intention; 67.8 % reported that they would seek help from formal sources at any time; and 42.7 % gave first priority to seeking help from formal sources. These percentages were smaller in the cases of irritability, insomnia, and depressed mood, and the smallest values were found in the case of irritability. McNemar test was performed to compare the percentages of participants reporting a positive help-seeking intention for each pair of health problems. Significant differences were found between all pairs of the health problems except the comparison between insomnia and depressed mood (*p* = 0.405).

**Table 3:** Intentions to seek help for four health problems

		Irritability		Dizziness		Insomnia		Depressed mood	
Help from formal sources	One of the choices	78	2.4 %	2244	67.8 %	1522	46.0 %	1269	38.4 %
	First choice	31	0.9 %	1414	42.7 %	827	25.0 %	620	18.7 %
Help from informal sources	One of the choices	2387	72.2 %	1808	54.7 %	1877	56.7 %	2041	61.7 %
	First choice	2379	71.9 %	1429	43.2 %	1668	50.4 %	1857	56.1 %
No help-seeking intention		898	27.1 %	465	14.1 %	813	24.6 %	831	25.1 %

Table 4 shows the comparisons of percentages of participants reporting a positive help-seeking intention. Most of the variables of interest showed significant associations with the help-seeking intention for all the health problems in univariate analyses.

**Table 4:** Percentages of subjects reporting a positive help-seeking intention

	Categories	N	Irritability			Dizziness			Insomnia			Depressed mood		
			*n*		*p*	*n*		*p*	*n*		*p*	*n*		*p*
Gender	Male	1621	988	61.0 %	<0.001	1274	78.6 %	<0.001	1129	69.6 %	<0.001	1090	67.2 %	<0.001
	Female	1687	1422	84.3 %		1569	93.0 %		1366	81.0 %		1387	82.2 %	
Age	20–29 years	797	558	70.0 %	0.057	649	81.4 %	<0.001	574	72.0 %	0.084	574	72.0 %	0.206
	30–39	842	638	75.8 %		728	86.5 %		644	76.5 %		638	75.8 %	
	40–49	837	616	73.6 %		741	88.5 %		643	76.8 %		635	75.9 %	
	50–59	832	598	71.9 %		725	87.1 %		634	76.2 %		630	75.7 %	
Marital status	Married	1960	1563	79.7 %	<0.001	1763	89.9 %	<0.001	1565	79.8 %	<0.001	1579	80.6 %	<0.001
	Unmarried	1184	737	62.2 %		937	79.1 %		805	68.0 %		776	65.5 %	
	Divorced/widowed	164	110	67.1 %		143	87.2 %		125	76.2 %		122	74.4 %	
Medical condition	No disease	2449	1788	73.0 %	0.116	2073	84.6 %	<0.001	1804	73.7 %	<0.001	1800	73.5 %	0.002
	Any disease	859	622	72.4 %		770	89.6 %		691	80.4 %		677	78.8 %	
Psychological well-being (WHO-5 score)	Low (0–12)	1707	1158	67.8 %	<0.001	1425	83.5 %	<0.001	1210	70.9 %	<0.001	1177	69.0 %	<0.001
	High (13+)	1601	1252	78.2 %		1418	88.6 %		1285	80.3 %		1300	81.2 %	
Psychiatric history	No	2689	1941	72.2 %	0.071	2268	84.3 %	<0.001	1987	73.9 %	<0.001	1975	73.4 %	<0.001
	Yes	619	469	75.8 %		575	92.9 %		508	82.1 %		502	81.1 %	
Contact with people with mental illness	No	2006	1335	66.6 %	<0.001	1617	80.6 %	<0.001	1404	70.0 %	<0.001	1400	69.8 %	<0.001
	Yes	1302	1075	82.6 %		1226	94.2 %		1091	83.8 %		1077	82.7 %	
Health literacy (HLS-14 score)	Low (14–50)	1853	1196	64.5 %	<0.001	1467	79.2 %	<0.001	1259	67.9 %	<0.001	1249	67.4 %	<0.001
	High (51+)	1455	1214	83.4 %		1376	94.6 %		1236	84.9 %		1228	84.4 %	
Attitude to mental illness 1	Yes	1613	1179	73.1 %	0.762	1408	87.3 %	0.030	1238	76.8 %	0.084	1213	75.2 %	0.677
(dangerous)	No	1695	1231	72.6 %		1435	84.7 %		1257	74.2 %		1264	74.6 %	
Attitude to mental illness 2	Yes	2171	1644	75.7 %	<0.001	1927	88.8 %	<0.001	1676	77.2 %	0.001	1675	77.2 %	<0.001
(unpredictable)	No	1137	766	67.4 %		916	80.6 %		819	72.0 %		802	70.5 %	
Attitude to mental illness 3	Yes	696	460	66.1 %	<0.001	558	80.2 %	<0.001	465	66.8 %	<0.001	471	67.7 %	<0.001
(blameworthy)	No	2612	1950	74.7 %		2285	87.5 %		2030	77.7 %		2006	76.8 %	
Attitude to mental illness 4	Yes	575	374	65.0 %	<0.001	455	79.1 %	<0.001	384	66.8 %	<0.001	383	66.6 %	<0.001
(never recover)	No	2733	2036	74.5 %		2388	87.4 %		2111	77.2 %		2094	76.6 %	
Social network (LSNS-6 score)	Low (0–11)	1891	1231	65.1 %	<0.001	1559	82.4 %	<0.001	1300	68.7 %	<0.001	1276	67.5 %	<0.001
	High (12+)	1417	1179	83.2 %		1284	90.6 %		1195	84.3 %		1201	84.8 %	
Neighborhood context 1	No	1221	744	60.9 %	<0.001	908	74.4 %	<0.001	778	63.7 %	<0.001	772	63.2 %	<0.001
(communicative)	Yes	2087	1666	79.8 %		1935	92.7 %		1717	82.3 %		1705	81.7 %	
Neighborhood context 2	No	2550	1779	69.8 %	<0.001	2144	84.1 %	<0.001	1858	72.9 %	<0.001	1837	72.0 %	<0.001
(trustful)	Yes	758	631	83.2 %		699	92.2 %		637	84.0 %		640	84.4 %	
Neighborhood context 3	No	2311	1584	68.5 %	<0.001	1919	83.0 %	<0.001	1662	71.9 %	<0.001	1634	70.7 %	<0.001
(helpful)	Yes	997	826	82.8 %		924	92.7 %		833	83.6 %		843	84.6 %	
Neighborhood context 4	No	2045	1354	66.2 %	<0.001	1664	81.4 %	<0.001	1430	69.9 %	<0.001	1404	68.7 %	<0.001
(cooperative)	Yes	1263	1056	83.6 %		1179	93.3 %		1065	84.3 %		1073	85.0 %	

Table 5 shows the results of multiple logistic regression analysis for the overall help-seeking intention. Perception of family and friends regarding help-seeking (their family and friends would think that they should receive help from others for the problem) showed the strongest association with the help-seeking intention for all the health problems. Besides this, the following factors were significantly associated with the help-seeking intention for all the health problems: female gender, unmarried status, psychiatric history, contact with people with mental illness, higher HLS-14 scores, higher LSNS-6 scores, and one of the neighborhood contexts (communicative).

**Table 5:** Factors related to overall help-seeking intention

	Category	Irritability		Dizziness		Insomnia		Depressed mood	
		OR	(95 % CI)	OR	(95 % CI)	OR	(95 % CI)	OR	(95 % CI)
Gender	Female	2.40	(1.99–2.89)	2.17	(1.68–2.81)	1.26	(1.04–1.53)	1.58	(1.31–1.91)
Age	plus 1 year	0.98	(0.97–0.99)	0.99	(0.99–1.01)	0.99	(0.98–1.00)	0.99	(0.98–1.00)
Marital status	Unmarried	0.58	(0.47–0.72)	0.71	(0.54–0.93)	0.75	(0.60–0.93)	0.63	(0.51–0.79)
Medical condition	Any disease	0.99	(0.80–1.24)	1.22	(0.90–1.67)	1.49	(1.18–1.89)	1.38	(1.09–1.73)
Psychological well-being (WHO-5 score)	plus 1 point	1.02	(1.01–1.04)	1.02	(0.99–1.04)	1.03	(1.01–1.05)	1.04	(1.03–1.06)
Psychiatric history	Yes	1.43	(1.11–1.84)	2.87	(1.94–4.25)	1.84	(1.40–2.41)	1.87	(1.43–2.45)
Contact with people with mental illness	Yes	1.72	(1.41–2.10)	2.02	(1.51–2.70)	1.36	(1.10–1.67)	1.38	(1.13–1.69)
Health literacy (HLS-14 score)	plus 1 point	1.04	(1.02–1.05)	1.04	(1.02–1.07)	1.03	(1.01–1.04)	1.02	(1.00–1.03)
Attitude to mental illness 1 (dangerous)	No	1.24	(0.99–1.55)	1.14	(0.84–1.54)	0.87	(0.69–0.90)	1.14	(0.90–1.42)
Attitude to mental illness 2 (unpredictable)	No	0.72	(0.57–0.90)	0.60	(0.45–0.82)	0.95	(0.75–1.20)	0.77	(0.61–0.98)
Attitude to mental illness 3 (blameworthy)	No	1.23	(0.97–1.54)	1.28	(0.95–1.71)	1.39	(1.10–1.76)	1.33	(1.05–1.67)
Attitude to mental illness 4 (never recover)	No	1.17	(0.92–1.50)	1.50	(1.10–2.05)	1.28	(0.99–1.65)	1.21	(0.94–1.55)
Social network (LSNS-6 score)	plus 1 point	1.06	(1.04–1.08)	1.04	(1.01–1.06)	1.06	(1.04–1.08)	1.06	(1.04–1.08)
Neighborhood context 1 (communicative)	Yes	1.24	(1.01–1.52)	2.22	(1.68–2.92)	1.38	(1.11–1.71)	1.26	(1.02–1.55)
Neighborhood context 2 (trustful)	Yes	1.32	(0.97–1.79)	1.06	(0.69–1.63)	1.11	(0.82–1.52)	1.21	(0.88–1.65)
Neighborhood context 3 (helpful)	Yes	0.88	(0.65–1.19)	0.82	(0.54–1.27)	0.89	(0.65–1.22)	0.95	(0.69–1.28)
Neighborhood context 4 (cooperative)	Yes	1.28	(0.99–1.64)	1.27	(0.89–1.81)	1.12	(0.87–1.45)	1.23	(0.95–1.58)
Perception of family and friends	Positive	3.37	(2.80–4.06)	5.07	(3.95–6.51)	5.04	(4.16–6.10)	3.97	(3.29–4.80)

*OR* adjusted odds ratio, *CI* confidence interval

Significant odds ratios (*p* < 0.05) are underlined

Table 6 shows the results of multiple logistic regression analysis for help-seeking intention from formal sources. Although the perception of family and friends was significantly associated with help-seeking intention from formal sources, the effect-sizes were lower than its association with overall help-seeking intention. Besides this, the following factors were significantly associated with the help-seeking intention for all the health problems: presence of any disease, psychiatric history, contact with people with mental illness, higher HLS-14 scores, one of the stigmatizing attitudes to mental illness (blameworthy), positive perception of the effectiveness of professional help, and one of the neighborhood contexts (communicative).

**Table 6:** Factors related to help-seeking intention from formal sources

	Category	Dizziness		Insomnia		Depressed mood	
		OR	(95 % CI)	OR	(95 % CI)	OR	(95 % CI)
Gender	Female	1.23	(1.04–1.46)	0.82	(0.70–0.96)	0.78	(0.66–0.91)
Age	plus 1 year	1.02	(1.01–1.03)	1.02	(1.01–1.03)	1.00	(0.99–1.01)
Marital status	Unmarried	1.09	(0.90–1.34)	1.28	(1.06–1.54)	1.06	(0.88–1.28)
Medical condition	Any disease	1.44	(1.17–1.79)	1.65	(1.37–1.99)	1.60	(1.33–1.92)
Psychological well-being (WHO-5 score)	plus 1 point	0.99	(0.97–1.00)	1.01	(0.99–1.02)	1.02	(1.01–1.04)
Psychiatric history	Yes	1.65	(1.29–2.10)	1.97	(1.60–2.43)	2.23	(1.82–2.75)
Contact with people with mental illness	Yes	1.66	(1.38–1.98)	1.58	(1.35–1.85)	1.42	(1.21–1.66)
Health literacy (HLS-14 score)	plus 1 point	2.09	(1.74–2.50)	1.54	(1.32–1.81)	1.52	(1.30–1.78)
Attitude to mental illness 1 (dangerous)	No	1.02	(0.84–1.25)	0.83	(0.69–0.99)	1.09	(0.91–1.31)
Attitude to mental illness 2 (unpredictable)	No	0.78	(0.63–0.96)	0.99	(0.82–1.21)	0.86	(0.71–1.05)
Attitude to mental illness 3 (blameworthy)	No	1.32	(1.07–1.64)	1.32	(1.08–1.62)	1.23	(1.00–1.52)
Attitude to mental illness 4 (never recover)	No	1.35	(1.08–1.70)	1.36	(1.09–1.70)	1.06	(0.85–1.32)
Perceived effectiveness of professional help	Positive	1.92	(1.62–2.27)	1.85	(1.57–2.18)	2.05	(1.73–2.43)
Social network (LSNS-6 score)	plus 1 point	1.01	(0.99–1.02)	1.01	(0.99–1.03)	1.01	(0.99–1.03)
Neighborhood context 1 (communicative)	Yes	1.73	(1.42–2.10)	1.29	(1.07–1.55)	1.27	(1.06–1.53)
Neighborhood context 2 (trustful)	Yes	0.82	(0.63–1.07)	1.10	(0.87–1.40)	1.15	(0.90–1.46)
Neighborhood context 3 (helpful)	Yes	0.83	(0.63–1.09)	0.78	(0.67–0.99)	0.81	(0.63–1.03)
Neighborhood context 4 (cooperative)	Yes	1.10	(0.88–1.38)	0.91	(0.75–1.11)	0.95	(0.78–1.16)
Perception of family and friends	Yes	2.41	(2.01–2.89)	2.68	(2.26–3.19)	2.05	(1.72–2.46)

*OR* adjusted odds ratio, *CI* confidence interval

Significant odds ratios (*p* < 0.05) are underlined

Table 7 shows the major reason for no help-seeking intention for the four health problems. Of the participants reporting no help-seeking intention, those who had hoped to receive help accounted for 35.7 % (321/898) for irritability, 23.9 % (111/465) for dizziness, 29.6 % (241/813) for insomnia, and 41.2 % (342/831) for depressed mood. The most frequently cited reason for no help-seeking intention for irritability was lack of awareness of potential sources of help. That for dizziness was willingness to handle the problem by oneself. For insomnia and depressed mood, negative perception of the effectiveness of help, lack of awareness of potential sources of help, and willingness to handle the problem by oneself were the three most frequent reasons for no help-seeking intention. On the other hand, when the reasons why they would avoid seeking help from formal sources were asked, the most frequently cited one was lack of awareness of potential sources of help for all of the problems of dizziness, insomnia, and depressed mood.

**Table 7:** Major reason for no help-seeking intention for four health problems

	Irritability		Dizziness		Insomnia		Depressed mood	
I feel no need to receive help so I will not.	577		354		572		489	
I hope to receive help but I will not, because.....	321		111		241		342	
I don’t have time.	7	2.2 %	1	0.9 %	5	2.1 %	5	1.5 %
There is no place to get appropriate help.	53	16.5 %	13	11.7 %	22	9.1 %	47	13.7 %
I am unsure where to go for help.	55	17.1 %	10	9.0 %	24	10.0 %	39	11.4 %
I don’t know how to get appropriate help.	1	0.3 %	0	0.0 %	2	0.8 %	1	0.3 %
The place is difficult to access.	0	0.0 %	1	0.9 %	0	0.0 %	1	0.3 %
I am concerned about the cost.	3	0.9 %	7	6.3 %	4	1.7 %	5	1.5 %
I think it hard to talk about such a personal problem.	27	8.4 %	8	7.2 %	15	6.2 %	32	9.4 %
People could not understand me.	16	5.0 %	7	6.3 %	22	9.1 %	41	12.0 %
I would not be satisfied with available help.	26	8.1 %	7	6.3 %	31	12.9 %	24	7.0 %
Available help would not do any good.	36	11.2 %	6	5.4 %	39	16.2 %	50	14.6 %
I am concerned about what people might think if I sought help.	13	4.0 %	5	4.5 %	3	1.2 %	8	2.3 %
I am afraid of revealing personal secrets.	15	4.7 %	2	1.8 %	3	1.2 %	6	1.8 %
I am afraid of being treated against my will.	0	0.0 %	1	0.9 %	1	0.4 %	0	0.0 %
I want to handle the problem on my own.	29	9.0 %	21	18.9 %	34	14.1 %	42	12.3 %
I think the problem will get better by itself.	7	2.2 %	7	6.3 %	16	6.6 %	14	4.1 %
Other	33	10.3 %	15	13.5 %	20	8.3 %	27	7.9 %

## Discussion

This study examined the help-seeking intentions for four kinds of health problems and their associated factors at the individual and neighborhood levels among Japanese adults. In contrast to previous studies that examined help-seeking intentions for specific mental disorders, [7, 8] this study focused on early signs that could appear before it is officially diagnosed as major depressive disorder. The four health problems presented in the questionnaire were designed so as to represent common signs and symptoms of depression. Our symptom-based approach may provide useful information for dealing with the problem of failure and delay in initial treatment contact after first onset of symptoms.

When the four health conditions indicated by irritability, dizziness, insomnia, and depressed mood were presented, the majority of participants recognized that help-seeking would be useful for solving the health problems and thus indicated their intentions to seek help. The greatest percentage of participants reporting a positive help-seeking intention was found for dizziness (physical problem), followed by insomnia (psychological problem), depressed mood (psychological problem), and irritability (non-medical problem). A similar result was obtained from the analysis for help-seeking intention from formal sources. These results indicated that psychological problems were less likely to induce help-seeking than a physical problem. Surprisingly, the likelihood of help-seeking for psychological problems was not greater than that for a non-medical problem. Cross-cultural comparative studies suggested that sociocultural factors influence symptom reporting in patients with depression; in particular, stigma surrounding mental illness increases the tendency to emphasize somatic symptoms [14, 52]. We infer from these findings that the difference in help-seeking intentions we observed in this study may be attributed to stigma surrounding mental illness. Many people probably considered dizziness as a symptom arising from physical causes, so that they may not have hesitated to seek help for this health problem.

Depressed mood and insomnia are included in the DSM-5 diagnostic criteria for major depressive disorder [26]. As a general rule in Japan, people who report these symptoms for over 2 weeks often screen positive for suspected depression. The depressed mood and insomnia problems of this study were intended to present health conditions that require prompt initial contact with healthcare providers. However, more than half of the participants did not choose medical professionals as a potential source of help for these health problems. This result supports the notion that many people with mental disorders tend not to receive professional help or use mental health services [53–56]. A negative perception of the effectiveness of help, a lack of awareness of potential sources of help, and willingness to handle the problem by oneself were the three most frequent reasons for no help-seeking intention. A lack of awareness of potential sources of help was also cited as the most common reason not to seek help from formal sources. In order to increase intentions to seek help from formal sources, policymakers should consider implementing universal interventions such as an education program [16] and an awareness campaign [57] which give all people a better understanding of potential benefits of professional help along with providing information on professional help available to the general public.

As for the depressed mood and insomnia problems, approximately half of the participants gave first priority to seeking help from informal sources, which were two to three times more than those who chose the options of seeking help from formal sources. Moreover, multiple logistic regression analysis revealed that perception of family and friends regarding help-seeking was significantly associated with the help-seeking intention from formal sources. As suggested in previous studies, [58, 59] family and friends may be regarded more favorably as helpful advisers, so that many people tend to first seek help from them. There is no doubt about the importance of having support from family and friends. However, family and friends may be both a positive and negative influence [24, 58, 59]. If the family and friends have inadequate knowledge about mental illness, their support could be unhelpful or even harmful. In order to increase prompt initial contact with healthcare providers, policymakers should consider improving support from family and friends. Public education campaigns to improve knowledge about mental illness may be adequate to this purpose [60]. Such universal interventions have the advantage of simultaneously targeting both affected individuals and their family and friends.

Multiple logistic regression analysis revealed that the factors significantly associated with help-seeking intentions were almost identical across the four health problems. This result can hardly explain the difference across types of health problems in the likelihood of help-seeking, but it supports the presence of factors associated with help-seeking intentions common to all problem types. In particular, perception of family and friends regarding help-seeking, psychiatric history, contact with people with mental illness, higher HLS-14 scores, and neighborhood communicativeness were significantly associated with the overall help-seeking intention and also the help-seeking intention from formal sources for all the problems of dizziness, insomnia, and depressed mood. Those who have had some exposure to mental illness are less likely to feel reluctant to seek help for mental illness [19–21]. Those who have better health literacy are more likely to acquire exact knowledge of mental illness and apply their knowledge to solving their health problems [44]. Those living in a neighborhood where neighbors say hello whenever they pass each other are more likely to receive the benefit of daily interactions with weak ties (i.e. peripheral members of social networks such as acquaintances) [61, 62]. People who often interact with weak ties, as well as strong ties (i.e. core members of social networks such as family and friends), are more likely to have a sense of belonging and thus less likely to hesitate to seek help from people around them. Besides developing health literacy skills, community-based interventions for creating a friendly approachable atmosphere and facilitating daily interactions with family, friends, and acquaintances may be worth considering as a possible public health strategy for encouraging help-seeking whether for psychological or physical problems. To date, several interventions that focus on facilitating social contact have been reported. For example, NOCOMIT-J is a community-based multimodal intervention program for suicide prevention in Japan, which set up regional social gatherings to reinforce human relationships in the community [57]. Further studies are needed to determine whether this kind of interventions can be recommended.

This study provides the first step toward understanding the types of health problems for which people intend to seek help. On the contrary, it has a number of potential limitations. First, the study participants were recruited from a nationwide panel of an online research company. As described in the methods section, the study participants included highly educated people twice as many as in the Japanese population. Although we confirmed that the distribution of HLS-14 scores in the study participants was quite similar to that obtained from our previous paper-based survey in Japanese healthcare facilities, [44] the selection bias may have influenced the results to some extent. Second, the web-based survey was self-administered, so that the accuracy of responses must depend on their understanding of questions and their motivation to answer questions accurately. Although the understandability of wording were checked prior to the web-based survey, it is almost impossible to eliminate the information bias completely. Third, the method of measuring help-seeking intentions was based on the most commonly used methodologies, [35–38] but its validity has not been fully confirmed. Respondents were asked to imagine themselves in specified hypothetical health conditions and then report their help-seeking intentions. If some people underestimated the severity of the health problem, the percentage of participants reporting a positive help-seeking intention in this study may have been somewhat different from the actual value. Fourth, the procedure of giving all four health problems to each participant may have affected participants’ responses. We cannot deny such influence, but the results of McNemar test indicated that those who thoughtlessly repeated the same responses across four health problems were few, if any. Fifth, because of the cross-sectional design, this study cannot provide definitive evidence of causality. There was no knowing whether the self-reported help-seeking intentions accurately reflect the actual help-seeking behaviors if they become mentally ill. Intention is recognized as a key predictor of behavior, but the strength of the intention-behavior relationship can vary depending on the type of behavior [63]. The findings of this study should be considered preliminary and need to be confirmed in other populations. In future studies, we intend to examine the relationship between intention to seek professional help and subsequent healthcare service use and elucidate in more detail the difference across types of health problems in the likelihood of help-seeking.

## Conclusions

The majority of participants indicated their intentions to seek help, but psychological problems (insomnia and depressed mood) were less likely to induce help-seeking than a physical problem (dizziness). A number of individual and neighborhood factors were significantly associated with help-seeking intentions across different problem types. In particular, perception of family and friends regarding help-seeking, psychiatric history, contact with people with mental illness, better health literacy, and neighborhood communicativeness were identified as the factors associated with help-seeking intentions common to all problem types. Besides developing health literacy skills, community-based interventions for creating a friendly approachable atmosphere and facilitating daily interactions with family, friends, and neighbors may be worth considering as a possible public health strategy for encouraging help-seeking whether for psychological or physical problems.

### Ethics and consent

The study protocol was approved by the ethics committee of the Jikei University School of Medicine and has been conducted in accordance with the Ethical Guidelines for Epidemiological Studies by the Japanese Government. Consent to participate was implied by the completion and submission of the survey.

### Consent for publication

There are no details on individual participants within the manuscript.

### Availability of data and materials

The dataset of this study will not be shared because the Ethical Guidelines prohibit researchers from providing their research data to other third-party individuals.

## Abbreviations

HLS-14: 14-item Health Literacy Scale
LSNS-6: abbreviated Lubben Social Network Scale
RIBS: Reported and Intended Behaviour Scale
WHO-5: World Health Organization Five Well-Being Index
YLD: year lived with disability

## References

[1] Global Burden of Disease Study 2013 Collaborators (2015). Global, regional, and national incidence, prevalence, and years lived with disability for 301 acute and chronic diseases and injuries in 188 countries, 1990-2013: a systematic analysis for the Global Burden of Disease Study 2013. Lancet.

[2] WHO World Mental Health Survey Consortium (2004). Prevalence, severity, and unmet need for treatment of mental disorders in the World Health Organization World Mental Health Surveys. JAMA.

[3] Wang PS, Angermeyer M, Borges G, Bruffaerts R, Tat Chiu W, DE Girolamo G, Fayyad J, Gureje O, Haro JM, Huang Y, Kessler RC, Kovess V, Levinson D, Nakane Y, Oakley Brown MA, Ormel JH, Posada-Villa J, Aguilar-Gaxiola S, Alonso J, Lee S, Heeringa S, Pennell BE, Chatterji S, Ustün TB (2007). Delay and failure in treatment seeking after first onset of mental disorders in the World Health Organization’s World Mental Health Survey Initiative. World Psychiatry.

[4] Jorm AF, Korten AE, Jacomb PA, Christensen H, Rodgers B, Pollitt P (1997). “Mental health literacy”: a survey of the public’s ability to recognise mental disorders and their belief about the effectiveness of treatment. Med J Aust.

[5] Jorm AF (2012). Mental health literacy: empowering the community to take action for better mental health. Am Psychol.

[6] Tylee A, Gandhi P (2005). The importance of somatic symptoms in depression in primary care. Prim Care Companion J Clin Psychiatry.

[7] Yap MB, Reavley N, Jorm AF (2013). Where would young people seek help for mental disorders and what stops them?: findings from an Australian national survey. J Affect Disord.

[8] Gibbons RJ, Thorsteinsson EB, Loi NM (2015). Beliefs and attitudes towards mental illness: an examination of the sex differences in mental health literacy in a community sample. PeerJ.

[9] Ikegami N, Yoo BK, Hashimoto H, Matsumoto M, Ogata H, Babazono A, Watanabe R, Shibuya K, Yang BM, Reich MR, Kobayashi Y (2011). Japanese universal health coverage: evolution, achievements, and challenges. Lancet.

[10] Mojaverian T, Hashimoto T, Kim HS (2013). Cultural differences in professional help seeking: a comparison of Japan and the u.s. Front Psychol.

[11] Jorm AF, Nakane Y, Christensen H, Yoshioka K, Griffiths KM, Wata Y (2005). Public beliefs about treatment and outcome of mental disorders: a comparison of Australia and Japan. BMC Med.

[12] Simon GE, VonKorff M, Piccinelli M, Fullerton C, Ormel J (1999). An international study of the relation between somatic symptoms and depression. N Engl J Med.

[13] Simon GE, Goldberg DP, Von Korff M, Ustün TB (2002). Understanding cross-national differences in depression prevalence. Psychol Med.

[14] Patel V (2001). Cultural factors and international epidemiology. Br Med Bull.

[15] Ono Y, Tanaka E, Oyama H, Toyokawa K, Koizumi T, Shinohe K, Satoh K, Nishizuka E, Kominato H, Nakamura K, Yoshimura K (2001). Epidemiology of suicidal ideation and help-seeking behaviors among the elderly in Japan. Psychiatry Clin Neurosci.

[16] Sakamoto S, Tanaka E, Neichi K, Ono Y (2004). Where is help sought for depression or suicidal ideation in an elderly population living in a rural area of Japan?. Psychiatry Clin Neurosci.

[17] Kido Y, Kawakami N (2013). Sociodemographic determinants of attitudinal barriers in the use of mental health services in Japan: findings from the World Mental Health Japan Survey 2002-2006. Psychiatry Clin Neurosci.

[18] Komiti A, Judd F, Jackson H (2006). The influence of stigma and attitudes on seeking help from a GP for mental health problems: a rural context. Soc Psychiatry Psychiatr Epidemiol.

[19] Jagdeo A, Cox BJ, Stein MB, Sareen J (2009). Negative attitudes toward help seeking for mental illness in 2 population-based surveys from the United States and Canada. Can J Psychiatry.

[20] Schomerus G, Matschinger H, Angermeyer MC (2009). The stigma of psychiatric treatment and help-seeking intentions for depression. Eur Arch Psychiatry Clin Neurosci.

[21] Mojtabai R (2010). Mental illness stigma and willingness to seek mental health care in the European Union. Soc Psychiatry Psychiatr Epidemiol.

[22] Rüsch N, Evans-Lacko SE, Henderson C, Flach C, Thornicroft G (2011). Knowledge and attitudes as predictors of intentions to seek help for and disclose a mental illness. Psychiatr Serv.

[23] ten Have M, de Graaf R, Ormel J, Vilagut G, Kovess V, Alonso J (2010). Are attitudes towards mental health help-seeking associated with service use? Results from the European Study of Epidemiology of Mental Disorders. Soc Psychiatry Psychiatr Epidemiol.

[24] Albert M, Becker T, McCrone P, Thornicroft G (1998). Social networks and mental health service utilization: a literature review. Int J Soc Psychiatry.

[25] Steele LS, Glazier RH, Lin E (2006). Inequity in mental health care under Canadian universal health coverage. Psychiatr Serv.

[26] Deneke DE, Schultz H, Fluent TE (2014). Screening for depression in the primary care population. Prim Care.

[27] Nakao M, Yano E (2006). Somatic symptoms for predicting depression: one-year follow-up study in annual health examinations. Psychiatry Clin Neurosci.

[28] Perlis RH, Fava M, Trivedi MH, Alpert J, Luther JF, Wisniewski SR, Rush AJ (2009). Irritability is associated with anxiety and greater severity, but not bipolar spectrum features, in major depressive disorder. Acta Psychiatr Scand.

[29] Verhoeven FE, Booij L, Van der Wee NJ, Penninx BW, Van der Does AJ (2011). Clinical and physiological correlates of irritability in depression: results from the Netherlands study of depression and anxiety. Depress Res Treat.

[30] Fava M, Hwang I, Rush AJ, Sampson N, Walters EE, Kessler RC (2010). The importance of irritability as a symptom of major depressive disorder: results from the National Comorbidity Survey Replication. Mol Psychiatry.

[31] Suka M, Yamauchi T, Sugimori H (2015). Relationship between individual characteristics, neighbourhood contexts and help-seeking intentions for mental illness. BMJ Open.

[32] Rickwood DJ, Deane FP, Wilson CJ (2007). When and how do young people seek professional help for mental health problems?. Med J Aust.

[33] Clement S, Schauman O, Graham T, Maggioni F, Evans-Lacko S, Bezborodovs N, Morgan C, Rüsch N, Brown JS, Thornicroft G (2015). What is the impact of mental health-related stigma on help-seeking? A systematic review of quantitative and qualitative studies. Psychol Med.

[34] Ministry of Internal Affairs and Communications. National Census 2010. Available at: http://www.e-stat.go.jp/SG1/estat/GL08020103.do?_toGL08020103_&tclassID=000001038689&cycleCode=0&requestSender=estat [Accessed 1 Oct 2015]. Japanese.

[35] Rickwood D, Thomas K, Bradford S. Help-seeking measures in mental health: a rapid review. August 2012 (Sax Institute Evidence Check Library). Available at: https://www.saxinstitute.org.au/publications/help-seeking-measures-in-mental-health-a-rapid-review [Accessed 1 Oct 2015]

[36] Wilson CJ, Deane FP, Ciarrochi J, Rickwood D (2005). Measuring help-seeking intentions: properties of the General Help-Seeking Questionnaire. Can J Couns.

[37] Oliver MI, Pearson N, Coe N, Gunnell D (2005). Help-seeking behaviour in men and women with common mental health problems: cross-sectional study. Br J Psychiatry.

[38] Reynders A, Kerkhof AJ, Molenberghs G, Van Audenhove C (2014). Attitudes and stigma in relation to help-seeking intentions for psychological problems in low and high suicide rate regions. Soc Psychiatry Psychiatr Epidemiol.

[39] Meurer WJ, Low PA, Staab JP (2015). Medical and psychiatric causes of episodic vestibular symptoms. Neurol Clin.

[40] World Health Organization Regional Office for Europe. Wellbeing Measures in Primary Health Care: the DepCare Project: Report on a WHO Meeting Stockholm, Sweden 12-13 February 1998. Copenhagen: WHO Regional Office for Europe; 1998.

[41] Awata S, Bech P, Yoshida S, Hirai M, Suzuki S, Yamashita M, Ohara A, Hinokio Y, Matsuoka H, Oka Y (2007). Reliability and validity of the Japanese version of the World Health Organization-Five Well-Being Index in the context of detecting depression in diabetic patients. Psychiatry Clin Neurosci.

[42] Evans-Lacko S, Rose D, Little K, Flach C, Rhydderch D, Henderson C, Thornicroft G (2011). Development and psychometric properties of the reported and intended behaviour scale (RIBS): a stigma-related behaviour measure. Epidemiol Psychiatr Sci.

[43] World Health Organization. Health promotion glossary, 1998 (WHO/HPR/HEP/98.1). Available at: http://www.who.int/healthpromotion/about/HPG [Accessed 1 Oct 2015].

[44] Suka M, Odajima T, Okamoto M, Sumitani M, Igarashi A, Ishikawa H, Kusama M, Yamamoto M, Nakayama T, Sugimori H (2015). Relationship between health literacy, health information access, health behavior, and health status in Japanese people. Patient Educ Couns.

[45] Suka M, Odajima T, Kasai M, Igarashi A, Ishikawa H, Kusama M, Nakayama T, Sumitani M, Sugimori H (2013). The 14-item health literacy scale for Japanese adults (HLS-14). Environ Health Prev Med.

[46] European Union. Mental well-being: special Eurobarometer 248/Wave 64.4, 2006. Available at: http://ec.europa.eu/health/ph_information/documents/ebs_248_en.pdf [Accessed 1 Apr 2015]

[47] DeVellis RF (2012). Scale development: theory and applications.

[48] Kessler RC, Ustün TB (2004). The World Mental Health (WMH) Survey Initiative Version of the World Health Organization (WHO) Composite International Diagnostic Interview (CIDI). Int J Methods Psychiatr Res.

[49] Lubben J, Blozik E, Gillmann G, Iliffe S, von Renteln Kruse W, Beck JC, Stuck AE (2006). Performance of an abbreviated version of the Lubben Social Network Scale among three European community-dwelling older adult populations. Gerontologist.

[50] Kurimoto A, Awata S, Ohkubo T, Tsubota-Utsugi M, Asayama K, Takahashi K, Suenaga K, Satoh H, Imai Y (2011). Reliability and validity of the Japanese version of the abbreviated Lubben Social Network Scale. Nihon Ronen Igakkai Zasshi.

[51] Tsuji I (2014). Health Survey of People Affected by the Great East Japan Earthquake in Miyagi Prefecture (MHLW Grant Research Report 2013).

[52] Bagayogo IP, Interian A, Escobar JI (2013). Transcultural aspects of somatic symptoms in the context of depressive disorders. Adv Psychosom Med.

[53] Jorm AF, Griffiths KM, Christensen H, Parslow RA, Rogers B (2004). Actions taken to cope with depression at different levels of severity: a community survey. Psychol Med.

[54] Rüdell K, Bhui K, Priebe S (2008). Do ‘alternative’ help-seeking strategies affect primary care service use?: a survey of help-seeking for mental distress. BMC Public Health.

[55] Brown J, Evans-Lacko S, Aschan L, Henderson MJ, Hatch SL, Hotopf M (2014). Seeking informal and formal help for mental health problems in the community: a secondary analysis from a psychiatric morbidity survey in South London. BMC Psychiatry.

[56] Andrade LH, Alonso J, Mneimneh Z, Wells JE, Al-Hamzawi A, Borges G, Bromet E, Bruffaerts R, de Girolamo G, de Graaf R, Florescu S, Gureje O, Hinkov HR, Hu C, Huang Y, Hwang I, Jin R, Karam EG, Kovess-Masfety V, Levinson D, Matschinger H, O’Neill S, Posada-Villa J, Sagar R, Sampson NA, Sasu C, Stein DJ, Takeshima T, Viana MC, Xavier M, Kessler RC (2014). Barriers to mental health treatment: results from the WHO World Mental Health surveys. Psychol Med.

[57] Ono Y, Awata S, Iida H, Ishida Y, Ishizuka N, Iwasa H, Kamei Y, Motohashi Y, Nakagawa A, Nakamura J, Nishi N, Otsuka K, Oyama H, Sakai A, Sakai H, Suzuki Y, Tajima M, Tanaka E, Uda H, Yonemoto N, Yotsumoto T, Watanabe N (2008). A community intervention trial of multimodal suicide prevention program in Japan: a novel multimodal community intervention program to prevent suicide and suicide attempt in Japan, NOCOMIT-J. BMC Public Health.

[58] Griffiths KM, Crisp DA, Barney L, Reid R (2011). Seeking help for depression from family and friends: a qualitative analysis of perceived advantages and disadvantages. BMC Psychiatry.

[59] Hui A, Wong P, Fu KW (2014). Building a model for encouraging help-seeking for depression: a qualitative study in a Chinese society. BMC Psychol.

[60] Dumesnil H, Verger P (2009). Public awareness campaigns about depression and suicide: a review. Psychiatr Serv.

[61] Granovetter M (1983). The strength of weak ties: a network theory revisited. Sociol Theory.

[62] Sandstrom GM, Dunn EW (2014). Social interactions and well-being: the surprising power of weak ties. Pers Soc Psychol Bull.

[63] Sheeran P (2002). Intention–behavior relations: a conceptual and empirical review. Eur Rev Soc Psychol.

